# Study of Guanxinning Injection on Regulatory Mechanism of Bcl-2 and Bax by Liquid Nitrogen Freezing-Mediated Femoral Head Necrosis

**DOI:** 10.1155/2017/4540124

**Published:** 2017-05-04

**Authors:** Xiaofeng Zhang, Xilin Xu, Gerhard Litscher, Zemin Sheng, Lu Wang, Daniela Litscher, Hang Lv, Xiaodong Li, Jie Zhang, Hui Su, Peiran Liu, Hai Hu

**Affiliations:** ^1^Heilongjiang University of Chinese Medicine, Harbin 150040, China; ^2^The Second Affiliated Hospital of Heilongjiang University of Chinese Medicine, Harbin 150001, China; ^3^Research Unit for Biomedical Engineering in Anesthesia and Intensive Care Medicine, Research Unit for Complementary and Integrative Laser Medicine and TCM Research Center Graz, Medical University of Graz, 8036 Graz, Austria; ^4^Privatclinic Lassnitzhoehe, 8301 Lassnitzhoehe, Austria

## Abstract

The objective of this study was to investigate the impact of femoral head perfusion by traditional Chinese medicine Guanxinning injection promoting blood circulation for removing blood stasis on the expression of Bcl-2 and Bax induced by liquid nitrogen freezing-mediated femoral head necrosis. 90 rabbits were randomized into three groups. Normal control group was not subjected to any medication. Saline and Guanxinning group were perfused with 0.9% saline and Guanxinning injection once every three days through the hip joint, respectively. Six animals in each group were sacrificed at weeks 1, 3, 6, 9, and 12. PCR and Western blot measured the expressions of Bcl-2 and Bax in the femoral head. The bax expression in the Guanxinning group reduced at the third week significantly compared to the normal control group (*P* < 0.01). The Bcl-2 expression in the Guanxinning group increased substantially at the third week (*P* < 0.05 or *P* < 0.01). Prolonged treatment elevated the expression of Bcl-2 in the Guanxinning group while that of Bax reduced remarkably (*P* < 0.01). Moreover, the ratio of Bcl-2 to Bax increased gradually in the Guanxinning group with prolonged drug administration. Guanxinning injection can inhibit the cell apoptosis of femoral head necrosis through the treatment by femoral head perfusion.

## 1. Introduction

Ischemic necrosis of femoral head is a frequently occurring disease, common in clinical orthopedics. It has a high morbidity, which is a major issue requiring immediate resolution in clinical practice [1, 2]. Although the artificial joint replacement, the only effective way of relieving pain and restoring joint range of motion [3], has achieved a significant progress, problems such as postoperative infection, prosthesis loosening, and prosthesis revision continue to persist. Moreover, many patients may experience several times of prosthesis revision during their lifetime [4], which is largely discomforting to the patients. Therefore, it is known as a catastrophic illness by some investigators [5]. Thus, head-preserving treatment has become the focus of current research. Presently, in the field of hip-preserving treatment of the femoral head necrosis, basic and clinical research of traditional Chinese medicine is widely applied; however, the drugs are restricted to the primary forms of the conventional peroral dosage form and topical plasters. Furthermore, there is no study regarding the characteristics and pathogenesis of the local incidence of femoral head necrosis, and the usage of local hip joint cavity perfusion of traditional Chinese medicine injection to treat the femoral head necrosis. Preclinical studies have shown that the local hip joint cavity perfusion of traditional Chinese medicine has a significant effect in treating femoral head necrosis [6, 7]. Angiogenesis is one of the major factors in the repair process of femoral head necrosis; blood activating and stasis removing drugs exhibit a precise effect in promoting angiogenesis [8] and are also the hot topics of research. Some studies have shown that the blood is activating, and stasis removing traditional Chinese medicine plays a positive regulatory role in the expression of the vascular endothelial growth factor (VEGF) [8, 9]. On the other hand, some studies have shown that [10] blood activating and stasis removing can inhibit the apoptosis of cardiomyocytes through multiple targets and reduce the index of apoptosis through multiple methods. Feng [11] found that promoting blood circulation compound can improve apoptosis and the expression of regulatory genes,* Bcl-2* and* Bax*. Our previous studies also found that hip joint cavity perfusion of Guanxinning injection can sustainably promote the expression of VEGF in femoral head necrosis [12] and effectively promote angiogenesis. However, whether it has a direct role in the formation of new bones and the resorption of necrotic bones in the repair process of femoral head necrosis is not known. Here, we aim to study the effect of femoral head perfusion of Guanxinning injection on apoptosis of femoral head necrosis induced by liquid nitrogen freezing.

## 2. Materials and Methods

### 2.1. Guanxinning Injection

Guanxinning injection (also known as Danshen Chuanxiong Injection) comprises extracts from two well-known traditional Chinese medicines, Danshen (*Salvia miltiorrhiza*) and Chuanxiong (Ligustrazine,* Ligustium Wallichii* Franch). Danshen and its active compounds tanshinones and isotanshinones have bioactivities against myocardial ischemia, inflammation, and angiotensin-converting enzyme. Chuanxiong and its active compounds tetramethylpyrazine and ferulic acid can dilate arteries, increase oxygen, and decrease platelet aggregation and thrombosis [13].

### 2.2. Experimental Animals

90 healthy 6-month-old New Zealand white rabbits (half males and half females) were selected. The average weight of the animals was 2.1 ± 0.1 kg. The animals were randomly divided into normal control group (30 animals) and modeling group (60 animals). After the animals with femoral head necrosis had been successfully modeled by liquid nitrogen freezing, they were again randomly divided into two groups, the saline group and the Guanxinning group (30 animals in each group).

The animals for the experimental purpose were provided by the Animal Center of Harbin Medical University, and the permit number was SCXK (HL) 2006-010. The rabbits were housed one animal/cage and fed for one week for adaptation. This study was approved by the Ethics Committee of The Second Affiliated Hospital of the Heilongjiang University of Chinese Medicine.

### 2.3. Reagents and Instruments

Guanxinning injection was purchased from Yuncheng Yinhu Pharmaceutical Co. Ltd. (Chinese Drug Approval Number: Z140219430). The following reagents and instruments were used in this study: anti-rabbit Bcl-2 antibody and anti-rabbit Bax antibody (Abcam); RNAiso plus, reverse transcription kit, and real-time polymerase chain reaction (PCR) kit (Takara, Japan); bicinchoninic acid (BCA) protein assay kit, gel preparation kit, and chemiluminescent (ECL) color developing reagent kit (Beyotime); real-time fluorescent quantitative PCR instrument (Roche 480 II); Lambda 25 ultraviolet spectrophotometer (Biochrom); high-speed freezing centrifuge (Hettich); and microplate reader (Tecan).

### 2.4. Modeling Methods

**Figure 1:**
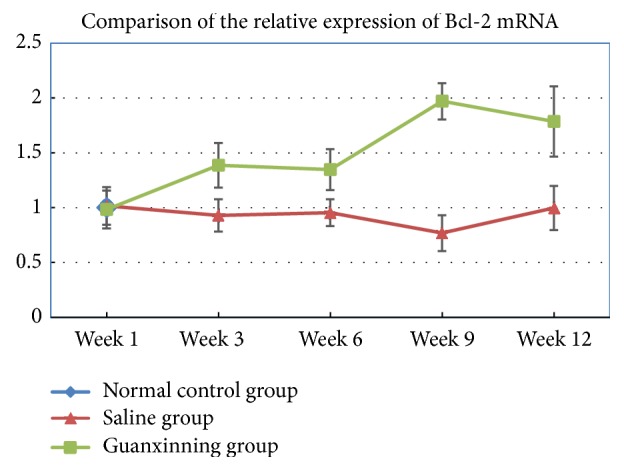
Expression of Bcl-2 mRNA of the femoral head in the normal control group, model group, and Guanxinning group at weeks 1, 3, 6, 9, and 12 after the treatment. (Expression of Bcl-2 mRNA in the normal control group at week 1 after the treatment is normalized to 1, and the legend shows the ratio of Bcl-2 mRNA in the normal control group to that in the other groups.)

Liquid nitrogen freezing was used to establish the rabbit model of femoral head necrosis [14]. After the animals had been intraperitoneally anesthetized with 30 mg/kg or 50 mg/kg pentobarbital, a 4 cm length of front external oblique incision of hip joint was selected. Subsequently, the skin, subcutaneous tissue, and deep fascia were incised sequentially, and anteromedial separation of the greater trochanter was exposed until the hip joint capsule. Then, the hip joint capsule was cut in a T-shaped manner to expose the femoral head, a crumpled gauze equal to the size of the femoral head was saturated with liquid nitrogen, and the upper end of the femoral head was frozen for 10 s. Finally, the upper end of the femoral head was then rewarmed using the warm saline gauze before reset, and each layer of the incision was sutured (Figure 1). After the operation, an intramuscular injection of 200,000 units of penicillin was administered twice daily in order to prevent wound infection.

### 2.5. Treatment Regimen

The normal control group was not subjected to any treatment. The saline group was treated with 0.9% saline (0.70 mL/times). The Guanxinning group was given Guanxinning injection (0.70 mL/times), and the hip joint perfusion was performed once every three days. The treatment started two weeks after the modeling operation, and the animals in each group were sacrificed at the postoperative time point of weeks 1, 3, 6, 9, and 12. At each time point, six animals were sacrificed for detection.

### 2.6. Experiment Procedure

According to the experimental steps, the animals were sacrificed after drug intervention. Each specimen was stored in liquid nitrogen for detection of Bcl-2 and Bax by real-time PCR and Western blot.

#### 2.6.1. Real-Time PCR

Real-time quantitative PCR detection was performed according to the instructions in the kit. Presuming that the amplification efficiency bias of the reference and target genes was within 5% and the amplification efficiency was nearly 100% for both genes, relative quantitative 2^−ΔΔCT^ was used to analyze the results. ΔCT of the target gene in each sample was obtained by subtracting the cycle threshold (CT) value of the reference gene from that of the target gene in each sample. ΔΔCT was obtained by subtracting the ΔCT of the normal control group from that of the treatment group. 2^−ΔΔCT^ was used to calculate the changes in the relative expression of the genes in each sample.

#### 2.6.2. Western Blot

Western blot was performed according to the following steps: extraction of the protein samples, estimation of the concentration of sample protein, sodium dodecyl sulfate polyacrylamide gel electrophoresis (SDS-PAGE), transfer to the membrane, immunoblotting, and chemiluminescence-based detection of the immunogens. The protein bands were assessed on X-ray films and images captured. The integrated optical density (IOD) value of each band was measured by ImageJ software.

### 2.7. Statistical Analysis

SPSS 19.0 statistical software was used to analyze the experimental results, which were represented as mean ± standard deviation. One-way ANOVA was used to compare data between the groups, and LSD test was used to perform multiple comparisons. *P* < 0.05 was considered to be statistically different, and *P* < 0.01 was considered to be distinctly different.

## 3. Results

### 3.1. Results of Real-Time PCR

#### 3.1.1. Expression of Bcl-2 mRNA

Low expression of Bcl-2 mRNA was detected in the normal control group. The expression was also less in the saline group at one week after administration. With prolonged administration, the expression further reduced slightly and was lower than the normal level. However, there was no difference when compared with the normal control group (*P* > 0.05). After the first week of administration, the expression in the Guanxinning group was not different from that in the normal control and the model groups (*P* > 0.05) but was distinctly different at the third week (*P* < 0.05 or *P* < 0.01); at the sixth week, they were significantly different (*P* < 0.01). In addition, the expression consistently increased until week 12 with statistically significant difference (*P* < 0.01) (Table 1).

#### 3.1.2. Expression of Bax mRNA

**Figure 2:**
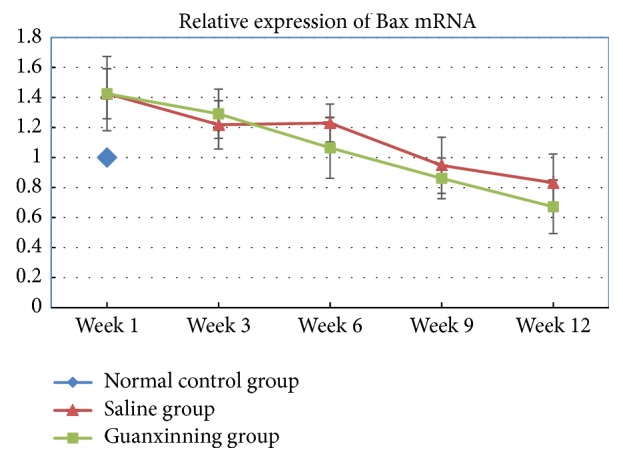
Expression of Bax mRNA of the femoral head in the normal control group, model group, and the Guanxinning group at weeks 1, 3, 6, 9, and 12 after the treatment. (Expression of Bax mRNA in the normal control group at week 1 after the treatment is normalized to 1, and the legend shows the ratio of Bax mRNA expression in the normal control group to that in the other groups.)

A difference in the expression of Bax mRNA in the normal control group during the different experimental stages was not observed. The expression of Bax mRNA in the saline group was significantly higher than the normal level at the early stage of weeks 1 and 3 (*P* < 0.05, *P* < 0.01). However, with a prolonged administration, its expression was gradually decreased until week 12, which was lower than the normal level (Figure 2). However, compared with the normal control group, there was no difference (*P* > 0.05). The Guanxinning group showed a high expression than the normal level at week 1 after the administration, with a distinct difference (*P* < 0.05 or *P* < 0.01) compared to the normal control or the saline groups. However, with an extended administration, the expression was gradually decreased until week 6, remarkably different when compared with the normal control group (*P* < 0.01). Moreover, at week 9, the expression decreased significantly when compared with the normal control and the saline groups (*P* < 0.01). Furthermore, it was lower than the normal level, which can constantly and steadily decrease until week 12 with a statistically significant difference (*P* < 0.01) (Table 2).

#### 3.1.3. Ratio of Bcl-2 mRNA to Bax mRNA

**Figure 3:**
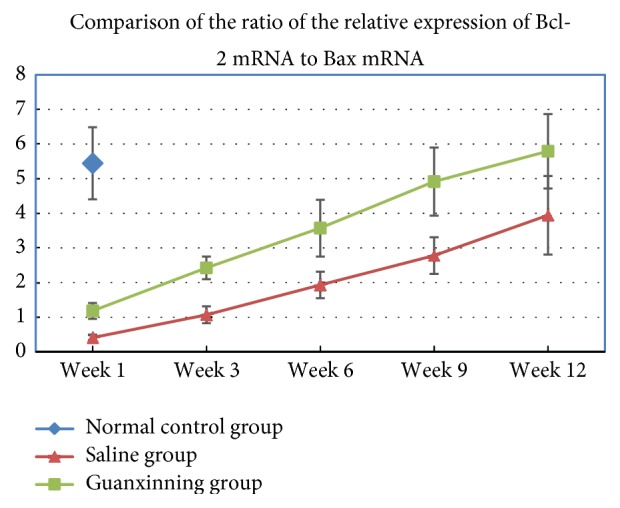
The ratio of Bcl-2 to Bax of the femoral head in the normal control group, model group, and the Guanxinning group at weeks 1, 3, 6, 9, and 12 after the treatment. (The ratio of Bcl-2 to Bax in the normal control group at week 1 after the treatment is normalized to 1, and the legend shows the ratios of Bcl-2 to Bax in this group and the other groups.)

A difference in the ratio of Bcl-2 to Bax at each time point of administration between the saline group and the normal control group was not observed (*P* > 0.05) (Figure 3). The ratio increased slightly in the Guanxinning group at week 1 after administration, but did not differ from that in the normal control and the saline groups. However, it increased markedly at week 3 compared to the normal control and saline groups (*P* < 0.05 or *P* < 0.01). With prolonged administration, the ratio constantly and steadily increased until week 6, with a significant difference compared with the normal control group and the saline group (*P* < 0.01). Moreover, the increase continued until week 12 (Table 3).

### 3.2. Relative Quantitative Analysis of Western Blot

#### 3.2.1. Comparative Analysis of the Relative Expression of Protein Bcl-2/*β*-Actin

**Figure 4:**
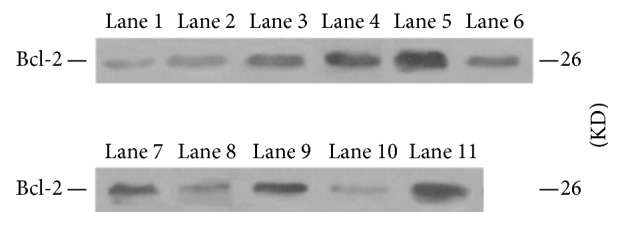
Expression of the Bcl-2 protein of the femoral head of rabbits in different groups at different time points. Lane 1 is the normal control group. Lane 2, lane 3, and lane 4 represent the model group, the control group, and the experimental group at week 1, respectively. Lane 5, lane 6, and lane 7 represent the model group, the control group, and the experimental group at week 3, respectively. Lane 8, lane 9, and lane 10 represent the model group, the control group, and the experimental group at week 6, respectively. Lane 11, lane 12, and lane 13 represent the model group, the control group, and the experimental group at week 9, respectively. Lane 14, lane 15, and lane 16 represent the model group, the control group, and the experimental group at week 12, respectively.

Bcl-2 protein was detected as 26 kDa band and the internal control *β*-actin as 41 kDa. In addition, the Bcl-2 bands were weak in each group (Figure 4). Bcl-2 was lowly expressed in the normal control group and minimally expressed in the saline group after week 1 of administration. However, with prolonged administration, the expression decreased slightly, which was lower than the normal level, and no difference was observed when compared with that in the normal control group. The expression in the Guanxinning group was not different from that in the normal control and saline groups at week 1 of administration (*P* > 0.05). Interestingly, the expression noticeably increased at week 3 and differed significantly when compared with the normal control group and the saline group (*P* < 0.05 or *P* < 0.01). Moreover, the difference was remarkable (*P* < 0.01) compared to week 6. With prolonged administration, the Bcl-2 expression constantly, steadily, and significantly increased until week 12 (*P* < 0.01). The protein level of Bcl-2 expression is illustrated in Figure 4 and Table 4.

#### 3.2.2. Comparative Analysis of the Relative Expression of Bax/*β*-Actin

**Figure 5:**
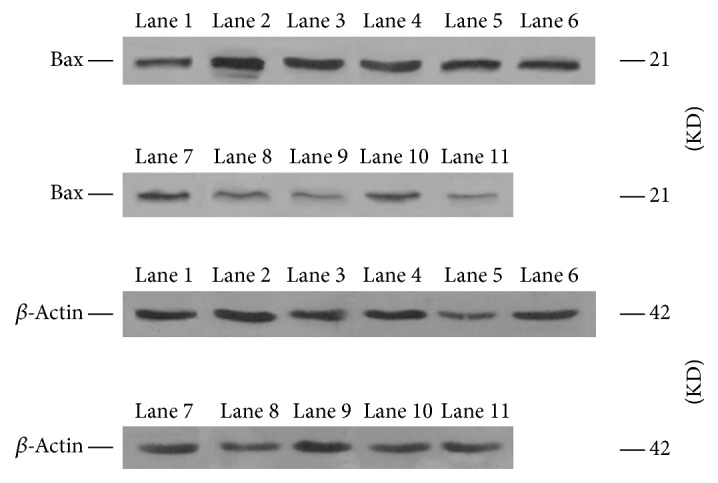
Expression of Bax protein of the femoral head of rabbits in different groups at various time points. Lane 1 is the normal control group. Lane 2, lane 3, and lane 4 are the model group, the control group, and the experimental group at week 1, respectively. Lane 5, lane 6, and lane 7 represent the model group, the control group, and the experimental group at week 3, respectively. Lane 8, lane 9, and lane 10 represent the model group, the control group, and the experimental group at week 6, respectively. Lane 11, lane 12, and lane 13 are the model group, the control group, and the experimental group at week 9, respectively. Lane 14, lane 15, and line 16 represent the model group, the control group, and the experimental group at week 12, respectively.

The Bax protein band appeared as 21 kDa, while the internal control *β*-actin was 42 kDa. The Bax protein bands were weak in each group (Figure 5). No change in the Bax expression was observed at different stages in the normal control group, as well as in the saline group at various stages; moreover, there was no difference when compared to the normal control group (*P* > 0.05). The expression was detected in the Guanxinning group at week 1 of administration, which was higher than the normal level, and an apparent difference was noted when compared with the normal control group (*P* < 0.05). With prolonged administration, the expression was gradually and distinctly decreased until week 9, when compared with the normal control and saline groups (*P* < 0.01 or *P* < 0.05). The lowest expression in the Guanxinning group that was significantly lower than the normal level appeared at week 12, which was significantly different from that in the normal control and the saline groups (*P* < 0.01). The protein level of Bax is shown in Figure 5 and Table 5.

#### 3.2.3. Ratio of the Relative Expression of the Protein of Bcl-2 to Bax

The ratio of the relative expression of Bcl-2 to Bax protein of the femoral head tissue in rabbits was significantly upregulated (Table 6). There was no change in the ratio of Bcl-2 to Bax in the normal control group at different stages. Moreover, the ratio of Bcl-2 to Bax in the saline group at various time points of administration did not vary significantly from that in the normal control group (*P* > 0.05). At week 1 of administration, the ratio of Bcl-2 to Bax in the Guanxinning group was increased slightly, which was slightly different from that in the normal control group (*P* < 0.05). The ratio was distinctly increased at week 3 and differed remarkably from that in the normal control and the saline groups (*P* < 0.05 or *P* < 0.01). With prolonged administration, the ratio can constantly and steadily increase until week 6, which was significantly different as compared to that in the normal control and the saline groups (*P* < 0.01); moreover, it steadily increased until week 12.

## 4. Discussion

Femoral head necrosis is a multipathological mechanism, eventually leading to multifactorial diseases of bone marrow cell ischemia and necrosis of bone cells. According to statistics, 100,000–150,000 new patients are presented with femoral bone necrosis every year in China [15], and the incidences are common in the younger population. Although the underlying mechanism of the incidence of femoral head necrosis is yet not clearly understood, some of its potential causes include alcoholism, usage of hormonal drugs, trauma, blood diseases, and radiation therapy [16]. Irrespective of the hormonal or alcoholic avascular necrosis of the femoral head, the process is not a simple cell death, rather a process that is involved in apoptosis [17–19]. During the repair process of the ischemic necrosis of femoral head, the prevention of apoptosis has become one of the main problems.

Apoptosis is a programmed cell death [20], as well as an important mechanism that maintains the body balance, and is regulated by a variety of genes, including* Bcl-2* (and its expression protein) that is known as an apoptosis suppressor gene [21]. Bcl2 was clarified in 1990 to possess an antiapoptotic function [22]. This gene can be expressed in the stimulation and development of a variety of normal cells, but it is neither expressed nor minimally expressed in mature or apoptotic cells. The Bcl-2 family includes critical regulatory factors in the pathways mediated by mitochondrial/cytochrome C, playing essential roles in controlling mitochondrial outer membrane permeabilization (MOMP), activating cytochrome C [23], and inhibiting apoptosis induced by multiple factors, including radiation, hormones, and growth factors. In addition, it can extend the time of cell survival via apoptosis impairment. In this study, Bcl2 was detected by real-time PCR and Western blot. In the Guanxinning group, the expressions of Bcl-2 protein and mRNA were detected at each time point. Bcl-2 showed a high expression at weeks 3 and 6 and was maintained until week 12, which was consistent with the above theory. It can be speculated that the traditional Chinese medicine involving blood activation and stasis removal can inhibit apoptosis of the local cells with ischemic necrosis of femoral head; the high expression of Bcl-2 can prolong the survival rate of cells and inhibit the apoptosis. Bax and Bcl-2 are homologous, and Bcl-2 has an antiapoptotic effect.* Bax* is an antagonistic gene of* Bcl-2*, and its biological effect is antagonistic on Bcl-2. The functional activity is carried out through its corresponding encoded functional protein, thereby inhibiting or promoting apoptosis. The impact of Bcl-2 on apoptosis is closely associated with the change in the ratio of Bcl-2 to Bax. The altered ratio determines whether the apoptosis or survival is a final destiny of the cells after stimulation by apoptotic signals. In the present study, the ratio of Bcl-2 to Bax was slightly increased in the Guanxinning group at week 1 after administration, and there was a slight difference compared with that in the normal control group (*P* < 0.05). With the extension of administration, the ratio constantly and steadily increased until week 12, and the difference was statistically significant (*P* < 0.01). Therefore, it can be speculated that Guanxinning injection can inhibit apoptosis to some extent in the apoptotic process of femoral head necrosis.

Several previous studies have shown that Bcl-2 regulates apoptosis through a combined action of multiple pathways, and its main mechanisms are as follows: (1) antagonistic gene* Bax*; (2) inhibition of proapoptotic cytochrome C entered into the cytoplasm after release from mitochondria; (3) prevention of cytochrome C in the cytoplasm to activate caspase; (4) antioxidant and maintenance of the homeostasis of intracellular calcium [24]. We speculated that a heterodimer was formed by the interaction and combination of Bax and Bcl-2 proteins. Then, the proapoptosis of Bax can be inhibited, which may be one of the vital regulatory mechanisms. In this study, the detection of Bcl-2 and Bax proteins, their mRNA expression, and altered ratio demonstrated that there was a weak Bax expression at the early stage in the bone tissue of femoral head in the saline group. Despite the saline intervention, with the extension of ischemic time, the expression showed a gradual increase to the peak in the first week, suggesting that the apoptosis might have appeared before the time point. Additionally, the ischemia continued until week 3, and there was a significant difference in Bax mRNA expression (*P* < 0.05 or *P* < 0.01) as compared to the normal control group. After week 3, the expressions of Bcl-2 mRNA and Bcl-2/*β*-actin were significantly increased, and there was a significant difference between the Guanxinning group and either the normal control group or the saline group (*P* < 0.05 or *P* < 0.01). However, with the extension of administration time, the expression of Bcl-2 mRNA and Bcl-2/*β*-actin in the Guanxinning group increased gradually, whereas the expression of Bax mRNA and Bax/*β*-actin was reduced gradually with a significant difference between the normal control and saline groups (*P* < 0.01). Moreover, we found that the ratio of Bcl-2/Bax, with prolonged administration time, gradually increased in the Guanxinning group, which was significantly higher than that in the saline group at each time point (*P* < 0.05). This result was in agreement with our speculation.

Taken altogether, we concluded that the Guanxinning injection could inhibit cell apoptosis of the femoral head necrosis through the treatment by femoral head perfusion.

## Figures and Tables

**Table 1:** Comparison of the relative expression of Bcl-2 mRNA of the femoral head tissue of rabbits in different groups (*X* ± SD, *n* = 6).

	Week 1	Week 3	Week 6	Week 9	Week 12
Normal control group	1.000 ± 0				
Saline group	1.016 ± 0.172	0.929 ± 0.148	0.954 ± 0.122	0.768 ± 0.163	0.997 ± 0.201
Guanxinning group	0.983 ± 0.172	1.387 ± 0.203^#▲▲^	1.347 ± 0.187^##▲▲^	1.971 ± 0.165^##▲▲^	1.786 ± 0.321^##▲▲^

Note: compared with the normal control group, # indicates *P* < 0.05, and ## indicates *P* < 0.01. Compared with the saline group at the same time point, ▲▲ indicates *P* < 0.01.

**Table 2:** Relative expression of Bax mRNA of the femoral head of rabbits in different groups at various time points (*X* ± SD, *n* = 6).

	Week 1	Week 3	Week 6	Week 9	Week 12
Normal control group	1.000 ± 0				
Saline group	1.426 ± 0.247^#^	1.218 ± 0.161^##^	1.230 ± 0.126	0.948 ± 0.187	0.832 ± 0.191
Guanxinning group	1.425 ± 0.166^##▲^	1.291 ± 0.164^##^	1.064 ± 0.203^##^	0.861 ± 0.136^##▲▲^	0.671 ± 0.178^##▲▲^

Note: compared with the normal control group at the same time point, # indicates *P* < 0.05, and ## indicates *P* < 0.01. Compared with the saline group at the same time point, ▲ indicates *P* < 0.05, and ▲▲ indicates *P* < 0.01.

**Table 3:** Comparison of the ratio of the relative expression of Bcl-2 mRNA to Bax mRNA of the femoral head of rabbits in different groups at various time points (*X* ± SD ± *s*).

Groups	Sample size	Week 1	Week 3	Week 6	Week 9	Week 12
Normal control group	6	5.446 ± 1.041				
Saline group	6	0.415 ± 0.082	1.072 ± 0.245	1.934 ± 0.382	2.781 ± 0.530	3.942 ± 1.136
Guanxinning group	6	1.183 ± 0.230	2.426 ± 0.331^#▲▲^	3.573 ± 0.817^##▲▲^	4.917 ± 0.982^##▲▲^	5.791 ± 1.073^##▲▲^

Note: compared with the normal control group at the same time points, # indicates *P* < 0.05, and ## indicates *P* < 0.01. Compared with the saline group at the same time point, ▲▲ indicates *P* < 0.01.

**Table 4:** Comparison of the relative expression of Bcl-2/*β*-actin protein of the femoral head of rabbits in different groups at different time points (*X* ± SD ± *s*).

Groups	Sample size	Week 1	Week 3	Week 6	Week 9	Week 12
Normal control group	6	0.537 ± 0.106				
Saline group	6	0.516 ± 0.148	0.531 ± 0.157	0.564 ± 0.182	0.603 ± 0.112	0.726 ± 0.135
Guanxinning group	6	0.683 ± 0.172	0.725 ± 0.135^#▲▲^	0.926 ± 0.231^##▲▲^	1.327 ± 0.244^##▲▲^	1.735 ± 0.122^##▲▲^

Note: compared with the normal control group at the same time point, # indicates *P* < 0.05, and ## indicates *P* < 0.01. Compared with the saline group at the same time point, ▲▲ represents *P* < 0.01.

**Table 5:** Comparison of the relative expression of Bax/*β*-actin protein of the femoral head of rabbits in different groups at various time points (*X* ± SD ± *s*).

Groups	Sample size	Week 1	Week 3	Week 6	Week 9	Week 12
Normal control group	6	0.895 ± 0.169				
Saline group	6	1.226 ± 0.217	1.114 ± 0.159	0.958 ± 0.204	0.987 ± 0.163	0.961 ± 0.178
Guanxinning group	6	1.324 ± 0.210^#^	1.072 ± 0.145^#^	0.824 ± 0.185^#^	0.732 ± 0.114^##▲^	0.570 ± 0.125^##▲▲^

Note: compared with the normal control group at the same time point, # represents *P* < 0.05, and ## represents *P* < 0.01. Compared with the saline group at the same time point, ▲ represents *P* < 0.05; ▲▲ represents *P* < 0.01.

**Table 6:** Comparison of the ratio of the relative expression of the proteins of Bcl-2 to Bax of the femoral head tissue in rabbits in different groups at various time points (*X* ± SD ± *s*).

Groups	Sample size	Week 1	Week 3	Week 6	Week 9	Week 12
Normal control group	6	2.741 ± 0.425	2.741 ± 0.425	2.741 ± 0.425	2.741 ± 0.425	2.741 ± 0.425
Saline group	6	0.343 ± 0.081	0.772 ± 0.134	1.608 ± 0.352	1.720 ± 0.257	1.937 ± 0.425
Guanxinning group	6	0.872 ± 0.204^#^	1.134 ± 0.204^#▲▲^	1.353 ± 0.421^##▲▲^	2.274 ± 0.482^##▲▲^	2.861 ± 0.537^##▲▲^

Note: compared with the normal control group, # represents *P* < 0.05, and ## represents *P* < 0.01. Compared with the saline group over the same period, ▲▲ represents *P* < 0.01.

## Abbreviations

BCA: Bicinchoninic acid
CT: Cycle threshold
IOD: Integrated optical density
MOMP: Mitochondrial outer membrane permeabilization
mRNA: Messenger ribonucleic acid
PCR: Polymerase chain reaction
SDS-PAGE: Sodium dodecyl sulfate polyacrylamide gel electrophoresis
VEGF: Vascular endothelial growth factor.
